# Fighting the Huntington’s Disease with a G-Quadruplex-Forming Aptamer Specifically Binding to Mutant Huntingtin Protein: Biophysical Characterization, In Vitro and In Vivo Studies

**DOI:** 10.3390/ijms23094804

**Published:** 2022-04-27

**Authors:** Claudia Riccardi, Federica D’Aria, Filomena Anna Digilio, Maria Rosaria Carillo, Jussara Amato, Dominga Fasano, Laura De Rosa, Simona Paladino, Mariarosa Anna Beatrice Melone, Daniela Montesarchio, Concetta Giancola

**Affiliations:** 1 Department of Chemical Sciences, University of Naples Federico II, 80126 Napoli, Italy; claudia.riccardi@unina.it; 2 Department of Pharmacy, University of Naples Federico II, 80131 Napoli, Italy; federica.daria@unina.it (F.D.); jussara.amato@unina.it (J.A.); 3 Research Institute on Terrestrial Ecosystems (IRET), UOS Naples-CNR, 80131 Napoli, Italy; filomenaanna.digilio@cnr.it (F.A.D.); mariarosaria.carillo@unicampania.it (M.R.C.); 4 Department of Experimental Medicine, University of Campania Luigi Vanvitelli, 80138 Napoli, Italy; 5 Department of Molecular Medicine and Medical Biotechnology, University of Naples Federico II, 80131 Napoli, Italy; dominga.fasano@gmail.com (D.F.); laura.derosa2@unina.it (L.D.R.); simona.paladino@unina.it (S.P.); 6 Center for Rare Diseases and Inter University Center for Research in Neurosciences, Department of Advanced Medical and Surgical Sciences, 2nd Division of Neurology, University of Campania Luigi Vanvitelli, 80131 Napoli, Italy; marina.melone@unicampania.it; 7 Center for Biotechnology, Sbarro Institute for Cancer Research and Molecular Medicine, Temple University, Philadelphia, PA 19122, USA

**Keywords:** G-quadruplex, aptamers, physico-chemical characterization, Huntington’s disease, *Drosophila melanogaster* model

## Abstract

A set of guanine-rich aptamers able to preferentially recognize full-length huntingtin with an expanded polyglutamine tract has been recently identified, showing high efficacy in modulating the functions of the mutated protein in a variety of cell experiments. We here report a detailed biophysical characterization of the best aptamer in the series, named MS3, proved to adopt a stable, parallel G-quadruplex structure and show high nuclease resistance in serum. Confocal microscopy experiments on HeLa and SH-SY5Y cells, as models of non-neuronal and neuronal cells, respectively, showed a rapid, dose-dependent uptake of fluorescein-labelled MS3, demonstrating its effective internalization, even in the absence of transfecting agents, with no general cytotoxicity. Then, using a well-established *Drosophila melanogaster* model for Huntington’s disease, which expresses the mutated form of human huntingtin, a significant improvement in the motor neuronal function in flies fed with MS3 was observed, proving the in vivo efficacy of this aptamer.

## 1. Introduction

Huntington’s disease (HD), also known as Huntington’s chorea, is an autosomal dominant inherited neurodegenerative disease characterized by a plethora of progressive motor, behavioural, cognitive, and psychiatric symptoms [1,2,3]. This debilitating disorder typically has midlife onset but can manifest at any time between infancy and senescence, showing mainly chorea and dystonia, incoordination, cognitive decline, and behavioural difficulties as predominant signs [4,5,6]. Despite the exceptional advances made in HD research, unfortunately, no definitive treatment is available for this invalidating disease, even if some potential therapeutics are in the pipeline [3,7,8,9,10,11].

Mutation in the first exon of *Huntingtin* (*HTT*) gene, which lies on the short arm of chromosome 4, has been identified as the main cause of HD disorder. This mutation consists of an abnormal repetition of the CAG triplet leading to the production of a mutant, misfolded HTT protein (mHTT) featured by a long polyglutamine tract (polyQ) [12,13]. This change in the amino acid sequence affects both the structure and physiological activity of the mutant protein. For example, the polyQ extension favours protein aggregation, especially in the caudate nucleus and putamen of basal ganglia, causing cortico-striatal dysfunction and degeneration [14,15,16]. In addition, polyQ expansion has a critical role in stimulating the physiological activity of methyltransferase polycomb repressive complex 2 (PRC2), which catalyses the trimethylation of histone H3 lysine 27 (H3K27me3), an epigenetic chromatin regulator [17,18].

Differences found between normal and mutant huntingtin suggested that molecules able to preferentially bind to the altered structure of the protein can be exploited as potential modulators of its activity. In this frame, oligonucleotide-based aptamers, being highly specific ligands, may offer a promising approach to slow down the progression of HD disease.

In detail, nucleic acid-based aptamers are short, single-stranded DNA or RNA molecules generally identified from large random oligonucleotide libraries using an in vitro selection procedure, known as SELEX (Systematic Evolution of Ligands by Exponential Enrichment) [19,20]. Upon folding into their specific three-dimensional arrangements, aptamers can specifically recognize with exceptionally high affinity and selectivity a wide range of different molecular targets, including proteins [21,22,23]. 

Various DNA- and RNA-based aptamers have been identified as valuable therapeutic candidates in several diseases [24,25,26,27,28], including neurodegenerative ones [29,30,31,32,33], and some examples have been recently reported for HD treatment [34,35,36,37,38,39,40].

In this context, four guanine-rich DNA-based aptamers—named MS1, MS2, MS3, and MS4, respectively—able to bind to the C-terminal CTD-II domain of a mutant huntingtin protein with an expanded 78-residue polyQ tract were recently identified by SELEX [41]. In this study, Shin and colleagues hypothesized that these aptamers could form G-quadruplex (G4) structures, which are non-canonical DNA or RNA architectures generated by the stacking of two or more guanine tetrads, i.e., cyclic planar arrangements of four guanines linked through Hoogsteen-type hydrogen bonds [42,43,44]. However, only preliminary Thioflavin T fluorescence-based assays were performed to support this structural hypothesis, and no data on their conformational behaviour and solution properties were provided. In addition, biological assays were carried out exclusively after aptamer transfection and no information was given on the cellular uptake of the aptamers in the absence of transfecting agents, as well as on their persistence in cells, that are crucial features for their bioactivity.

Among the investigated oligonucleotides, the strongest binder proved to be MS3, carrying the sequence d(GGGAGGGAGGGAGGGAGGGAGGGAGGGAGGGAGGGA). This aptamer was found to co-localize with endogenous mutant huntingtin in neuronal progenitor cells (NPCs). In addition, it proved to decrease PRC2 activity, and its transfection significantly increased ATP levels protecting NPCs against starvation-dependent stress [41].

Considering the high therapeutic potential in HD of MS3 and aiming at providing a deep insight into its physico-chemical features, we herein investigated the conformational behaviour of this aptamer, along with its thermodynamic stability, nuclease resistance in vitro and biological activity in vitro and in vivo [41].

## 2. Results and Discussion

### 2.1. MS3 Aptamer Adopts a Stable, Parallel G-Quadruplex Structure: A Physico-Chemical Characterization

#### 2.1.1. UV Spectroscopy Analysis

Buffer composition, and especially cation type and concentration, play fundamental roles in G-quadruplex formation, by influencing the peculiar folding topology adopted by a selected G-quadruplex-forming oligonucleotide [45,46,47].

In this study, the spectroscopic properties and conformational behavior of MS3 were investigated in two different buffer solutions, one exclusively containing sodium ions as cations—i.e., 10 mM NaH_2_PO_4_/Na_2_HPO_4_, 90 mM NaCl, indicated as Na^+^-containing buffer—and the other containing small amounts of potassium ions so as to mimic pseudo-physiological buffers (i.e., PBS: 137 mM NaCl, 2.7 mM KCl, 10 mM NaH_2_PO_4_/Na_2_HPO_4_, 1.8 mM KH_2_PO_4_/K_2_HPO_4_, pH 7.3).

We firstly validated the ability of MS3 to fold into a G-quadruplex structure in the selected Na^+^-rich solutions by UV spectroscopic studies.

In detail, UV thermal difference spectra (TDS, Figure S1) were obtained, recording spectra at 2 μM oligonucleotide concentration at low and high temperatures (i.e., 5 and 100 °C) in both the tested saline conditions. The UV spectra difference between the unfolded (at 100 °C) and folded (at 5 °C) oligonucleotide represents indeed a “fingerprint” of a specific nucleic acid structure and can be used to confirm the formation in solution of a G-quadruplex structure [48].

The normalized TDS profiles of MS3 were similar in the examined buffer solutions, with two positive (at ca. 240 and 275 nm) and two negative bands (around 260 and 295 nm), which are diagnostic of a G-quadruplex structure (Figure S1), according to the literature [48].

In addition, an estimation of the predominant G-quadruplex conformation in solution was obtained by determining the TDS factors [49]. In both the analyzed saline conditions, the ΔA_240_/ΔA_295_, ΔA_255_/ΔA_295_, and ΔA_275_/ΔA_295_ factors provided values higher than 4, 3.5, and 4, respectively (Table S1), which were all consistent with a parallel G-quadruplex folding, in accordance with literature data [49].

#### 2.1.2. CD and DSC Analyses

Circular dichroism (CD) and differential scanning calorimetry (DSC) analyses were exploited as complementary methodologies to derive information on the conformational behaviour and thermodynamic stability of the G-quadruplex structure formed by MS3 in solution. Indeed, these techniques allow determination of the melting temperature (T_m_) value and the energy needed to unfold the native structure of MS3.

In detail, circular dichroism is a powerful, quick methodology for the determination of the topology and thermal stability of G-quadruplexes [50,51].

**Figure 1:**
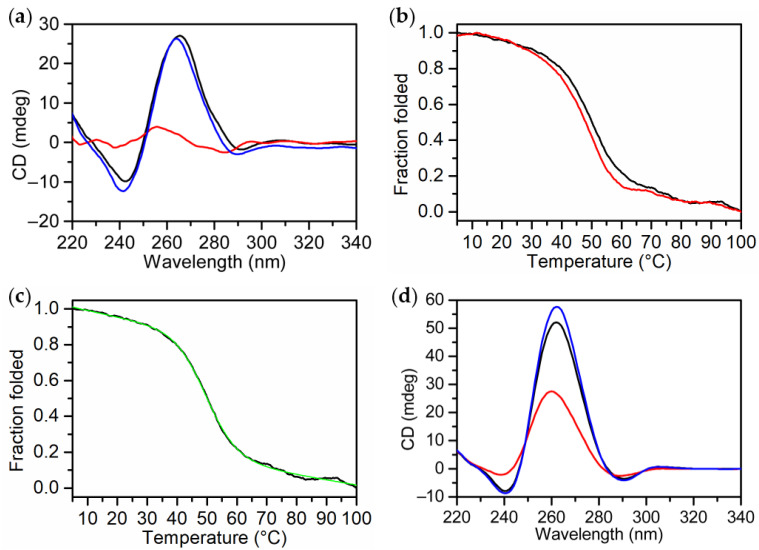
CD analysis of MS3 at 2 µM in the Na^+^-containing buffer (10 mM NaH_2_PO_4_/Na_2_HPO_4_, 90 mM NaCl solution, pH 7.0): (**a**) CD spectra of MS3 at 5 °C before (black line), after melting/cooling (blue line) and at 100 °C (red line); (**b**) CD melting (black line) and cooling (red line) profiles of MS3 recorded at 0.5 °C min^−1^; (**c**) CD melting curve (black line) and calculated Van ’t Hoff curve (green line). (**d**) CD spectra of MS3—recorded at 2 µM in PBS buffer (137 mM NaCl, 2.7 mM KCl, 10 mM NaH_2_PO_4_/Na_2_HPO_4_, 1.8 mM KH_2_PO_4_/K_2_HPO_4_, pH 7.3)—at 5 °C before (black line), after melting/cooling (blue line), and at 100 °C (red line).

CD spectra of pre-annealed solutions of MS3 were first collected at 5 °C in both the selected Na^+^-rich solutions (Figure 1a,d; black lines). In all cases, they showed the characteristic profile of a parallel G-quadruplex structure with a maximum at 263 nm and a minimum at 240 nm [52,53,54]. Notably, positive CD bands showed higher intensities in PBS than in the Na^+^-containing solution, indicating a higher structuration degree in the first buffer, according to the well-known ability of K^+^ to stabilize G4 structures better than Na^+^ ions [45,46,47] and the overall higher ionic strength of PBS vs. the chosen Na^+^-containing solution.

In order to definitively confirm the G4 structure topology adopted by MS3 in the examined solutions, CD spectra recorded at 5 °C were also processed by singular value decomposition (SVD) analysis, using a suitable software developed by del Villar-Guerra and coworkers [55]. This analysis provided fitted CD profiles in very good accordance with the experimental CD curves (Figure S2), indicating that MS3 exclusively adopts a parallel G4 conformation in both buffers, also in line with the calculated TDS factor values.

CD-monitored thermal denaturation experiments were then carried out on MS3 dissolved in the Na^+^-containing buffer following CD signal changes at 263 nm as a function of temperature in the 5–100 °C range (Figure 1b). In this saline condition, the melting and cooling curves of MS3 were almost superimposable and showed no hysteresis, indicating reversible heating/cooling processes (Figure 1b). Different temperature gradients (0.5 and 1 °C min^−1^) were also tested, obtaining similar results, thus demonstrating that the unfolding/refolding process is not kinetically controlled (data not shown). In this buffer, the CD spectra of MS3 at 5 °C before and after a heating/cooling cycle were essentially superimposable (Figure 1a, black and blue lines, respectively), further corroborating the reversibility of the unfolding/refolding process, and the aptamer proved to be fully denatured at 100 °C (Figure 1a, red line). Thus, by applying the two-state model Van ’t Hoff analysis to the CD melting curve (Figure 1c, green line), a T_m_ value of 50 °C and a Δ_vH_H° value of 167 kJ mol^−1^ were determined. 

The CD analysis of MS3 in PBS showed that in this buffer solution, the aptamer was folded in a much more stable G-quadruplex structure which, although recovering almost completely the initial spectral features after the melting/cooling cycle (Figure 1d, blue line), was not fully denatured even at 100 °C (Figure 1d, red line).

**Figure 2:**
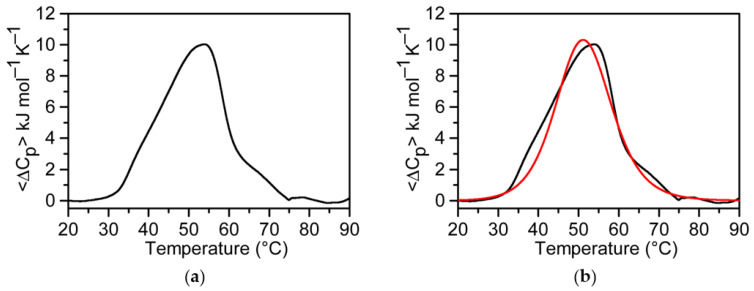
DSC analysis of MS3 in the Na^+^-containing buffer (10 mM NaH_2_PO_4_/Na_2_HPO_4_, 90 mM NaCl solution, pH 7.0). (**a**) Experimental DSC profile (black line), and (**b**) Van ’t Hoff calculated curves based on the two-states model (red line) superimposed on the experimental curve (black line).

For a deeper insight into the thermal stability of MS3 G-quadruplex structure, DSC measurements were also performed. A detailed thermodynamic analysis of MS3 could be carried out only in the Na^+^-containing buffer, since the absence of a complete unfolding of the G-quadruplex structure formed by MS3 in PBS, even at high temperatures (cfr. Figure 1d), prevented an accurate calorimetric study in the latter buffer. The DSC profile of MS3 in the selected Na^+^-containing solution is shown in Figure 2a. The melting temperature as well as the enthalpy and entropy change values were directly obtained from the experimental DSC curve without any model assumption and are reported in Table 1.

The DSC data clearly showed that the denaturation of the G-quadruplex structure is an entropy-driven process. This favourable entropy change, as a result of the gain in conformational freedom degrees of the oligonucleotide upon unfolding, is compensated by an unfavourable enthalpy contribution, due to the loss of G-tetrad stacking interactions. Considering that the Δ_exp_H° value per tetrad typically falls in the range 60–80 kJ mol^−1^, as calculated taking into account the experimental values of a large number of G-quadruplexes depending on molecularity (unimolecular, bimolecular, tetramolecular), cations (Na^+^ or K^+^) and presence of loops (short or long) [56,57,58,59], it can be deduced that the G-quadruplex structure of MS3 is stabilized by three guanine tetrads, corresponding to a Δ_exp_H° value per tetrad of ~68 kJ mol^−1^.

Figure 2b shows the comparison between the experimental and calculated DSC curves, based on the two-state Van ’t Hoff equation. The calculated profile does not perfectly match the experimental DSC profile. The calculated Van ’t Hoff enthalpy change (Δ_vH_H°) is 175 kJ mol^−1^, close to the value obtained by using the CD melting profile. The ratio Δ_exp_H°/Δ_vH_H° is higher than one, suggesting the presence of significantly populated intermediate(s) [60].

To check the presence of intermediate species, three-dimensional melting curves for MS3 were obtained by collecting whole CD spectra as a function of temperature, every two degrees, with a scan rate of 0.5 °C min^−1^ (Figure S3). The T_m_ value determined from the analysis of 3D melting curves is in perfect agreement with those obtained at a single wavelength at 0.5 °C min^−1^ scan rate. The 3D melting curves were submitted for single value decomposition (SVD) analysis to verify the number of significant spectral species involved in the equilibrium melting experiments [61]. SVD analysis showed that three components must be considered when analyzing the data sets derived by melting experiments (Table S2), confirming the presence of an intermediate state during the unfolding process, which thus well explains the observed difference between the Δ_exp_H° and the Δ_vH_H° data.

### 2.2. MS3 Aptamer Is Highly Resistant to Nuclease Degradation

The resistance of oligonucleotides to nuclease degradation occurring in serum is one of the most crucial parameters determining their potential in vivo use. 

**Figure 3:**
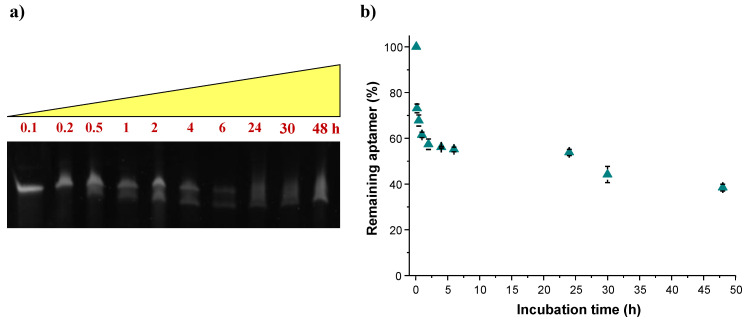
Enzymatic resistance experiments performed on MS3 incubated in 80% fetal bovine serum (FBS) as monitored by 20% denaturing polyacrylamide gel electrophoresis up to 48 h (time points: 0.1, 0.2, 0.5, 1, 2, 4, 6, 24, 30 and 48 h). (**a**) Representative 20% denaturing PAGE (8 M urea). Samples were loaded at 3 μM concentration, and the gel was run at a constant 200 V at r.t. for 3.5 h in TBE 1× as running buffer. (**b**) Time-dependent degradation of MS3 in the presence of FBS. The intensity of the band corresponding to the intact oligonucleotide on the gel is expressed as percentage of the remaining intact aptamer with respect to that of the first monitored point and reported as a function of the incubation time. Data are reported as mean values ± SD (error bars) for multiple determinations.

Aiming at evaluating the stability to enzymatic digestion of MS3, this oligonucleotide—previously dissolved and annealed in PBS—was incubated in 80% (*v*/*v*) fetal bovine serum (FBS) at 37 °C and its integrity was monitored up to 48 h. Samples withdrawn from these reaction mixtures at fixed times, as indicated in Figure 3, were then analyzed by gel electrophoresis under denaturing conditions following the previously described procedures [62,63]. The intensity of each oligonucleotide band on the gel was then calculated and expressed as a normalized percentage with respect to that of the first monitored point, taken 2 min after incubation in FBS (Figure 3b).

PAGE experiments revealed a slow reduction in the DNA band intensity up to 24 h, where the remaining intact oligonucleotide was 50% of the initial amount. Indeed, starting from 30 min of monitoring time, an additional band with faster mobility was observed, in accordance with the expected fragmentation of MS3, forming shorter oligonucleotide species. Notably, the aptamer did not completely disappear even after 48 h incubation in FBS, when MS3 was still detectable at least for ca. 40% of its initial amount.

These results proved that MS3 is a highly stable aptamer in serum, which makes it potentially suitable for in vivo studies. Its good resistance to nucleases well correlates with its ability to fold into a very stable G-quadruplex structure, as evidenced by both spectroscopic and calorimetric data. Overall, these results indicate that MS3 adopts a very compact three-dimensional G-quadruplex folding, with limited regions exposed to the solvent, which protects the oligonucleotide backbone from rapid enzymatic degradation.

### 2.3. MS3 Aptamer Is Efficiently Internalized in Different Cell Types in Concentration- and Time-Dependent Manner and Persists for a Long Time

**Figure 4:**
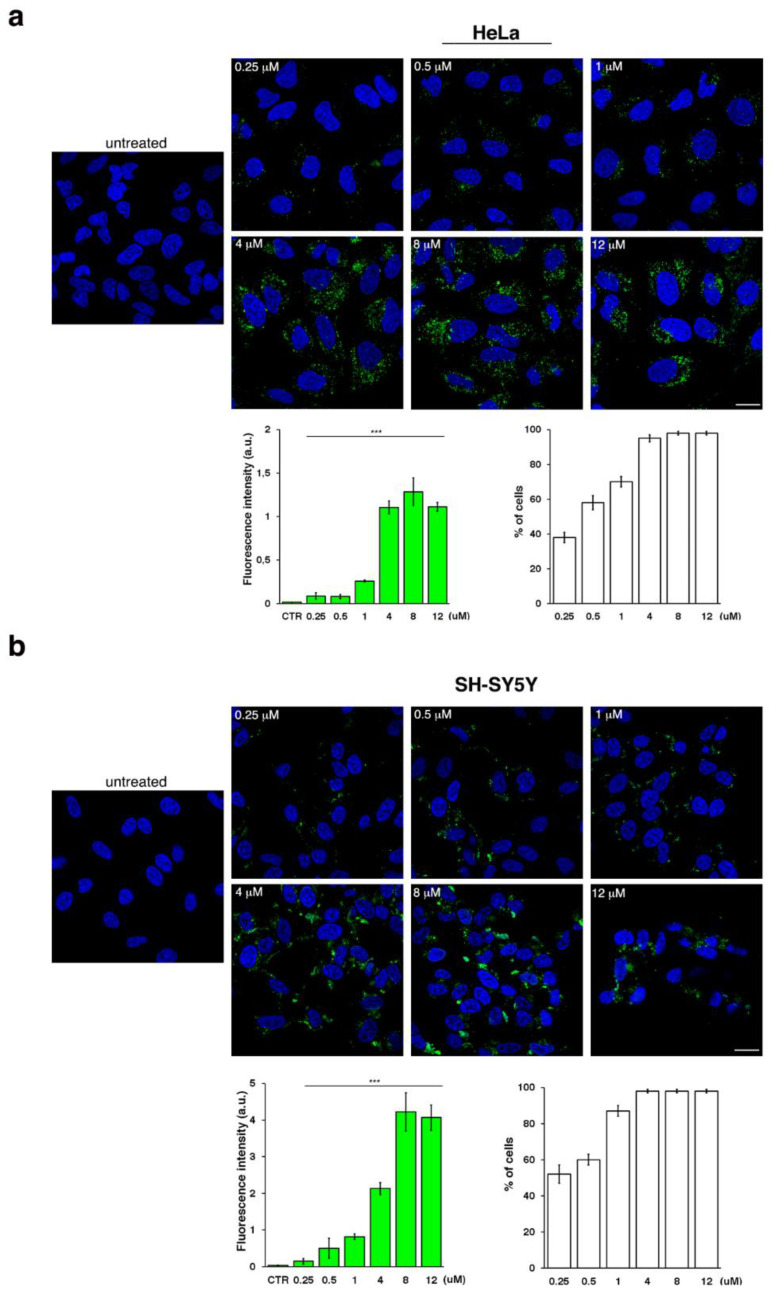
FITC-MS3 uptake in different cell lines. HeLa (**a**) or SH-SY5Y (**b**) cells were incubated with FITC-conjugated MS3 (green) for 24 h at different concentrations, as indicated. Then, cells were fixed, and nuclei were stained with DAPI (blue). Images were acquired with a confocal microscope. Scale bars, 6 µM. Mean fluorescence intensity normalized per cell area (arbitrary unit, a.u.) of three independent experiments is shown (left graph), *n* > 50 cells; error bars, mean ± SD. *** *p* < 0.0001, Student *t*-test. The percentage of fluorescent cells, measured as number per field, is shown (right graph); error bars, mean ± SD. Note that the percentage of fluorescent cells increased in dose-dependent manner (*p* < 0.001, Student *t*-test), reaching a plateau at the concentration of 4 µM (no statistical significance among 4, 8, 12 µM, Student *t*-test).

To evaluate the biological activity of MS3 and its potential use in the treatment of HD, we first analyzed its cellular uptake. To this aim, we selected two human cell lines, i.e., HeLa and neuroblastoma-derived SH-SY5Y cells, as models of non-neuronal and neuronal cells, respectively. Both cell lines were incubated for 24 h with different concentrations of fluorescein isothiocyanate (FITC)-conjugated MS3. Fluorescent signals were detected both in HeLa and SH-SY5Y cells (Figure 4), indicating that this aptamer was efficiently internalized in both the tested cell lines. The rate of cellular uptake increased in a dose-dependent manner, as clearly shown by the quantitative analysis (Figure 4, bar graph on the left), thus indicating that MS3 uptake is a concentration-dependent process in both the examined cell types. Moreover, the number of cells displaying a fluorescent signal was proportional to the MS3 concentration. At low concentrations, about 35–50% of cells gave a detectable fluorescence signal; starting from 4 µM, almost all cells proved to incorporate the aptamer (Figure 4, bar graph on the right). 

**Figure 5:**
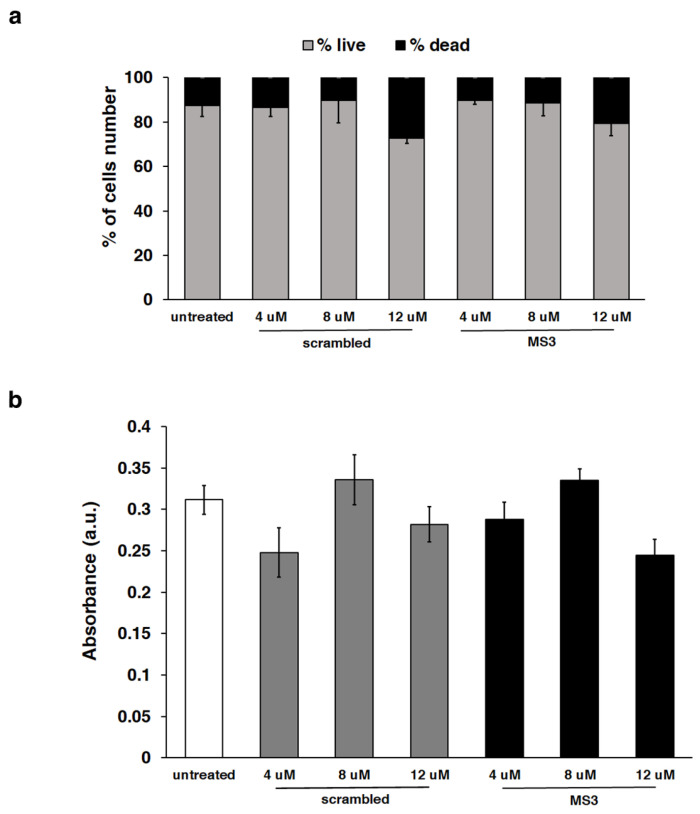
Evaluation of cell viability and metabolic activity of SH-SY5Y cells after treatment with the scrambled oligonucleotide or MS3 aptamer in comparison with untreated cells. (**a**) Percentage of viable and dead cells after trypan blue staining. (**b**) MTT assay results. For both assays, the reported values represent the mean of biological triplicate of two independent experiments; error bars, mean ± SD. No statistical significance, Student *t*-test.

Importantly, no evident effects of cytotoxicity were detected, as determined by monitoring both cell lines by morphological analysis (Figure S4) and appearance of nuclei stained with DAPI (Figure 4). In addition, considering the intrinsic higher sensitivity of neuronal cell types to exogenous agents, we also evaluated the cell viability by trypan blue assays and the metabolic activity by MTT assays for SH-SY5Y cells (Figure 5). The percentage of live/dead cells and metabolic activity proved to be comparable in cells treated with the MS3 aptamer or with a scrambled oligonucleotide, used as a negative control, at all the tested concentrations. Remarkably, no significant difference was observed comparing treated with untreated cells (Figure 5), thus supporting the conclusion that MS3 does not have relevant cytotoxic effects on the tested cells.

**Figure 6:**
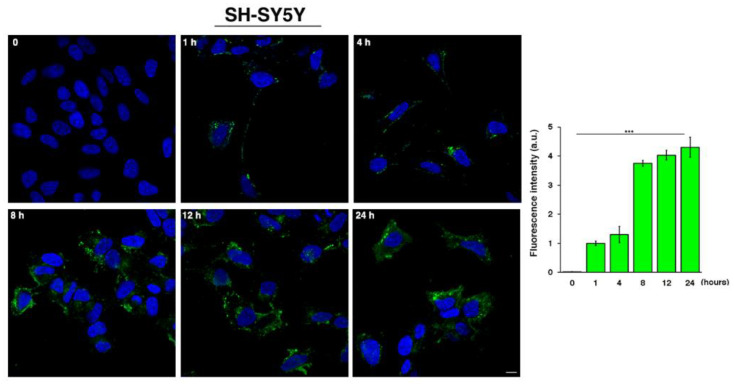
Time-course analysis of MS3 cell uptake. SH-SY5Y cells were incubated with a 4 µM solution of FITC-MS3 (green) at the indicated time points and fixed; then, nuclei were stained with DAPI (blue). Images were acquired with confocal microscope. Scale bar, 6 µm. Mean fluorescence intensity normalized per cell area (arbitrary unit, a.u.) of three independent experiments is shown (left graph), *n* > 50 cells; error bars, mean ± SD. *** *p* < 0.0001, Student *t*-test.

Successively, time-course experiments of the aptamer cell uptake were performed by incubating SH-SY5Y cells with a 4 µM solution of FITC-MS3 (chosen for being the minimum concentration with a good uptake rate) at different time points. MS3 was found to be quickly internalized in the cells as early as 1 h (as evident from a significant fluorescent signal) and reached maximum values between 12 and 24 h (Figure 6).

**Figure 7:**
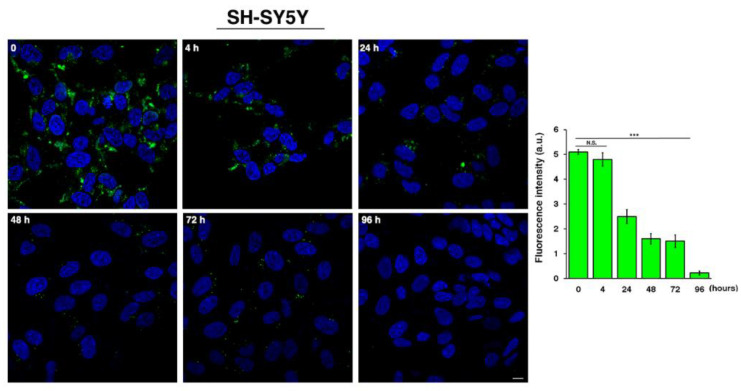
Analysis of MS3 biological stability by time-course experiments. SH-SY5Y cells were incubated for 24 h with FITC-MS3 (green). After removal of residual aptamers by extensive washings, cells were incubated in culture medium for the different indicated times. Nuclei were counterstained with DAPI. Images were acquired with confocal microscope. Scale bar, 6 µm. Mean fluorescence intensity normalized per cell area (arbitrary unit, a.u.) of three independent experiments is shown (left graph), *n* > 40 cells; error bars, mean ± SD. *** *p* < 0.0001, Student *t*-test; N.S., not significant.

To evaluate the persistence/stability of MS3 in the cells, SH-SY5Y cells were incubated with FITC-MS3 (4 µM) for 24 h (time 0), washed to remove the unincorporated MS3, and then incubated for different time periods in the culture medium (Figure 7). Interestingly, MS3 persisted in the cells at least until 72 h, although the fluorescent signal progressively decreased (Figure 7), overall indicating good stability and compatibility with cell life.

Overall, the efficiency of MS3 cell uptake and its long persistence within cells, along with the absence of detectable cytotoxic effects, proved to be favourable features of this aptamer in vitro, which constitute a solid basis to advance its potential use for in vivo therapeutic strategies.

### 2.4. MS3 Aptamer Improves Motor Neuronal Function in a Drosophila Huntington’s Disease Model

To evaluate the effects of MS3 aptamer in vivo, we studied the influence of dietary MS3 on locomotor activity of HD flies, as a parameter of improved health. To this aim, we used a well-established *Drosophila melanogaster* model for HD (Q128HD-FL), which expresses the mutated human HTT protein, containing 128 glutamine repeats in the exon 1, in all neuronal tissues (genotype: elav-Gal4/+; UAS-HttFL-Q128/+) [64]. These Q128HD-FL transgenic flies replicate most key hallmarks of the HD neuronal dysfunction, including decreased lifespan, progressive accumulation of aggregates in the cytoplasm, age-dependent locomotor impairment, and experience a very aggressive course of HD disease. An initial hyperactivity is followed by quickly progressive age-dependent motor dysfunction and coordination difficulties due to impaired motor neuronal function [65,66].

In these experiments, a statistically significant number of Q128HD-FL transgenic flies were fed with six different MS3 concentrations: a control diet devoid of MS3, and five “Assay fly Food” (AF, see Section 3) media, each supplemented with different concentrations of the MS3 aptamer (1.5, 3.5, 6.25, 12.5 and 25 μM). First, to be sure of the MS3 palatability, we monitored adult feeding behavior by adding the red food dye no. 40 to AF medium for each MS3 concentration used. The amount of food consumed in one day was estimated by examining the fly abdominal coloring under a stereomicroscope. We found that Q128HD-FL transgenic adult flies fed with both the MS3 at different doses and the food coloring did not display variation in food intake, showing the same intensity of body coloring after visual evaluation.

Then, to test whether the MS3 oral treatment was able to slow down the course of the disease and the onset of pathological symptoms, adult motor function was measured as the ability of Q128HD-FL flies to climb a vial wall as a function of MS3 assumption at days 1, 3, 6 and 9 post-eclosion.

**Figure 8:**
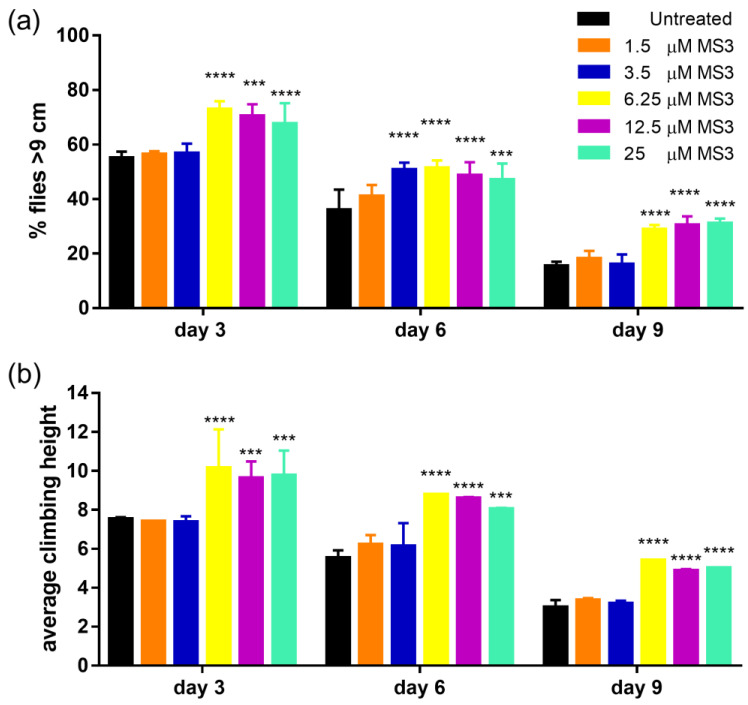
Climbing ability of age-matched adult female flies grown on food supplemented with different doses of MS3, evaluated at four different points: presymptomatic (pre-HD; 1 day post-eclosion, not shown), early symptomatic (early-HD; 3 days post-eclosion) and late symptomatic (late-HD; 6 and 9 days post-eclosion). (**a**) Percentage of treated flies that climbed over 9 cm was higher in comparison to sibling flies in a dose-dependent manner; (**b**) average climbing height reached by treated and untreated flies, both in pre- and late symptomatic periods. For each condition, the climbing ability of three groups of 20 flies was monitored (*n* = 60) for a total of 3 trials (*n* = 180). Analysis of data was conducted using ANOVA one way; data represents mean ± SEM (**** *p* < 0.0001; *** *p* < 0.001; compared with untreated as control).

We calculated the percentage of flies climbing over 9 cm (Figure 8a) and the average climbing height reached (Figure 8b). Our results clearly show that MS3 treatment significantly improved the climbing ability of Q128HD-FL transgenic flies in a dose-dependent manner. At the lowest concentrations used (1.5 and 3.5 μM), we did not observe any effect on the climbing ability of Q128HD-FL flies. As shown in Figure 8a,b, motor dysfunction of flies treated with 1.5 and 3.5 μM MS3 and of untreated flies was similar; it was early and progressive and on day 3 post-eclosion, more than 50% of flies could not reach the 9 cm target. In contrast, the climbing ability of flies fed with higher concentrations of the MS3 aptamer (i.e., 6.25, 12.5 and 25 μM) was significantly higher. At day 3 post-eclosion, about 70% of flies could reach the 9 cm target. Their motor disability arose later and was most prominent on day 9 post-eclosion, when, in contrast, untreated flies tended to stay at the bottom of the vial.

In conclusion, both the percentage of flies that reached the target (9 cm) and the average climbing height reached was significantly higher at each examined age point (*p* < 0.0001), indicating that MS3 slowed down the progressive loss of motor function and the onset of this pathological symptom in a dose-dependent manner. Dietary administration of doses higher than 6.25 μM of MS3 suppresses motor dysfunction in flies expressing Q128HD-FL. The 6.25 μM concentration seemed to be the minimum concentration of MS3 that was effective in improving motor dysfunction in Q128HD-FL fly model. 

Our findings strongly suggest that MS3 ameliorates polyQ-induced compromised neuronal function in Q128HD-FL flies and, in consideration also of the absence of toxic effects, not observed either in vitro or in vivo, can be a promising candidate for the development of innovative drugs to reduce the effects of HD disease. 

## 3. Experimental Section

### 3.1. Materials 

Acrylamide/bis-acrylamide (19:1) 40% solution, glycerol, formamide, urea and GelGreen Nucleic Acid Stain were purchased from VWR (Milan, Italy). Ammonium persulfate (APS) and tetramethylethylenediamine (TEMED) were purchased from Sigma Aldrich (Merck Life Science, Milan, Italy). Fetal bovine serum (FBS) was provided by Euroclone (Pero, Milan, Italy).

### 3.2. Oligonucleotide Sample Preparation

MS3 aptamer [d(GGGAGGGAGGGAGGGAGGGAGGGAGGGAGGGAGGGA)], 5′-FITC-modified MS3 and a scrambled oligonucleotide of the same length as MS3, unable to form G-quadruplex structures, [d(GTGAGTGAGTGAGTGAGTGAGTGAGTGAGTGAGTGA)], were purchased from Biomers.net GmbH (Ulm, Germany) as HPLC-purified sequences. All the used oligonucleotides were characterized by MALDI and proved to be >97% pure by HPLC analysis, as provided by the manufacturer.

The concentration of each oligonucleotide was evaluated by UV measurements at 260 nm at a temperature of 95 °C, using molar extinction coefficient values calculated by the nearest-neighbour model [67]. For the biophysical characterization, unmodified MS3 was analyzed either in sodium phosphate buffer (10 mM NaH_2_PO_4_/Na_2_HPO_4_, 90 mM NaCl, pH 7.0) or in PBS (137 mM NaCl, 2.7 mM KCl, 10 mM NaH_2_PO_4_/Na_2_HPO_4_, 1.8 mM KH_2_PO_4_*/*K_2_HPO_4_, pH = 7.3). The solutions were heated at 90 °C for 5 min and then slowly cooled to room temperature.

### 3.3. UV Spectroscopy Analysis

UV spectra were acquired on a JASCO V-770 UV–Vis spectrophotometer equipped with a Peltier Thermostat JASCO ETCS-761, by using a quartz cuvette with a 1 cm path length (1 mL internal volume, Hellma). MS3 was dissolved in the selected phosphate buffers so to obtain 2 μM solutions and then slowly annealed. In detail, absorbance spectra were recorded at 5 and 100 °C in the range 220–320 nm using a scanning speed of 100 nm min^−1^ and subtracting the proper baseline. Then, thermal difference spectra (TDS) were obtained by subtracting the UV spectrum recorded at 5 °C, at which the aptamer is fully structured, from the one obtained at 100 °C, where the G-quadruplex structure is completely denatured [48,49]. UV measurements at each temperature were carried out in duplicate.

In order to facilitate the comparison of the spectral data, all the obtained TDS profiles were then normalized to the maximum of absorbance simply by dividing the raw data by the maximum absorbance value, so that the highest positive peak has a Y-value of +1 [48]. From normalized spectra, TDS factors (ΔA_240_/ΔA_295_, ΔA_255_/ΔA_295_, and ΔA_275_/ΔA_295_) were also determined in both the analyzed buffer solutions as the ratios between the absolute absorbance values at different wavelengths [48,49].

### 3.4. Circular Dichroism (CD) Analysis

CD experiments were carried out on a Jasco J-815 spectropolarimeter (JASCO Inc., Tokyo, Japan) equipped with a PTC-423S/15 Peltier temperature controller, or on Jasco J-1500 spectropolarimeter equipped with a Jasco CTU-100 circulating thermostat unit, using a quartz cuvette with a path length of 1 cm. All the spectra were recorded at 5 °C in the 220–340 nm wavelength range and averaged over three scans. The scan rate was 100 nm min^−1^, with a 4 s response and 1 nm bandwidth. Oligonucleotide concentration was 2 μM for all the CD samples. CD melting and cooling curves were recorded in the 5–100 °C range at 0.5 or 1 °C min^−1^ by following changes of the CD signal at the wavelength of maximum intensity (263 nm). Three-dimensional melting curves were obtained by recording the CD spectra as a function of temperature using the same parameters reported above. CD spectra were recorded every two degrees. The CD melting curves at 263 nm were fitted by a two-state transition equation according to the Van ’t Hoff analysis using Origin 7.0 software (OriginLab Corp., Northampton, MA, USA). The melting temperature (T_m_) and enthalpy change (∆_vH_H°) values provide the best fit of the experimental melting data. All experiments were performed in duplicate, and the reported values were the average of two measurements.

For the singular value decomposition (SVD) analysis performed on the CD spectra acquired at 5 °C, the obtained spectra were treated so as to convert the *Y*-axis unit from mdeg to molar ellipticity, as previously reported [68]. The resulting spectra were then processed as reported in the literature [55].

Data from CD thermal denaturation/renaturation profiles were also converted into folded fraction, as previously described [69,70].

### 3.5. Differential Scanning Calorimetry (DSC) Analysis

DSC measurements were carried out on a nanoDSC (TA Instruments, New Castle, DE, USA), using a 200–400 µM G4 sample dissolved in the 10 mM NaH_2_PO_4_/Na_2_HPO_4_, 90 mM NaCl solution at pH 7.0. The apparent molar heat capacity vs. temperature profiles were obtained at 0.5 °C min^−1^ scan rate. The excess heat capacity function 〈∆C_P_°〉 was obtained after buffer-buffer baseline subtraction. No baseline difference was observed before and after the transition, indicating a negligible heat capacity difference between the initial and final states. The experimental enthalpy change, ∆_exp_H°, was obtained by integrating the area under the 〈∆C_P_°〉 vs. temperature curve and the entropy change, ∆_exp_S°, was obtained by integrating the area under the 〈∆C_P_°〉/T versus temperature curve. The T_m_ value was obtained from the maximum of each DSC curve. The Gibbs energy change, Δ_exp_G°, was calculated at 37 °C, from the equation Δ_exp_G° = Δ_exp_H° − 310.15·Δ_exp_S°. The reported thermodynamic parameters were the average of at least three independent heating experiments.

### 3.6. Singular Value Decomposition Analysis (SVD)

Three-dimensional melting curves were analyzed by singular value decomposition (SVD) to evaluate the number of significant spectral species during the transition. SVD was performed using Matlab R2020b software (The MathWorks Inc., Natick, MA, USA). The matrix of the CD spectra A is decomposed into product matrices U, S, and V by SVD (A = U·S·V^T^). S is a diagonal matrix that contains the singular values. U is a matrix of basis spectra for each spectral component. The V matrix consists of amplitude vectors for each basis spectrum, corresponding to a spectral component change with temperature. The autocorrelation function of the basis spectra and amplitude vectors represents a measure of nonrandom shapes within each column and allows determining the minimum number of component spectra required to describe the data. A value of the autocorrelation functions higher than 0.6 was selected as a cutoff criterion for accepting a significant spectral species.

### 3.7. Enzymatic Stability Assays Monitored by Gel Electrophoresis Analysis

The MS3 stability in serum was determined by gel electrophoresis analysis according to reported procedures [62,63], with minor modifications. Briefly, MS3—previously annealed in PBS buffer at 50 μM conc.—was incubated in 80% FBS at 37 °C. Then, at fixed times, 3 μL of the samples (corresponding to 30 pmol) were collected, mixed with formamide (1:2, *v*/*v*) to immediately quench the enzymatic degradation, heated at 95 °C for 5 min, and finally stored at −20 °C until subsequent analysis. Thereafter, all the samples—supplemented with 5% glycerol immediately before loading—were analyzed by gel electrophoresis on 20% denaturing PAGE using 8 M urea in TBE 1X as running buffer. The gels were run at r.t., at constant 200 V for 3.5 h, then stained with GelGreen Nucleic Acid Stain (supplemented with 0.1 M NaCl) for 30 min and finally visualized with a UV transilluminator (BioRad ChemiDoc XRS, Milan, Italy). The experiment was repeated 3 times. The intensity of the DNA bands on the gel, at each collected time, was then calculated by using the FiJi software and normalized with respect to the first monitoring point. Percentages of the remaining intact oligonucleotide are reported as mean values ± SD for multiple determinations. 

### 3.8. Cell Cultures

HeLa and SH-SY5Y cells were maintained in RPMI-1640 (Euroclone, Pero, Italy) with 10% fetal bovine serum (FBS; Hyclone, Fisher Scientific, Waltham, MA, USA), and 2 mM L-glutamine (Euroclone, Pero, Italy). All cell lines were maintained at 37 °C in a saturated humidity atmosphere containing 95% air and 5% CO_2_.

### 3.9. Cytotoxic Assays

#### 3.9.1. MTT Assay

To evaluate cell viability, the selected cell lines were seeded in 24-well plates at 5 × 10^4^ cells per well and incubated with the scrambled oligonucleotide or MS3 aptamer as above described. After 72 h, cells were washed with RPMI and incubated with 3-[4,5-dimethylthiazol-2-yl]-2,5-diphenyltetrazolium bromide (MTT) solution (0.5 mg/mL; Sigma-Aldrich, St Louis, MO, USA) for 1 h at 37 °C under 5% CO_2_. Then, the MTT assay media were discarded. The crystals of formazan were dissolved using 500 μL of dimethyl sulfoxide (DMSO) and the resulting coloured solution was analyzed by measuring the absorbance values at 595 nm with a Microplate Reader according to the manufacturer’s protocol. 

#### 3.9.2. Trypan Blue Assay

Cells were trypsinized and diluted 1:1 with trypan blue stain (10 μL cells and 10 μL of 0.4% trypan blue stain). The viable and dead cells were differentiated by trypan blue exclusion and counted by Corning Cell Counter (Corning Inc., New York, NY, USA) using CytoSMART™ software (CytoSMART Technologies, Eindhoven, The Netherlands). 

### 3.10. Fluorescence Microscopy

To monitor MS3 aptamer internalization, FITC-MS3 was added to the cells in culture medium at 37 °C at different concentrations or for different time periods, as indicated. Then, cells were washed with PBS and fixed with 4% paraformaldehyde (PFA), quenched with 50 mM NH_4_Cl. Images were collected using confocal laser scanning microscope LSM 700 (Carl Zeiss, Jena, Germany) equipped with a Plan Apo 63 X oil immersion objective (NA 1.4). Diode lasers at 405 and 488 nm were used as light source; fluorescence emission was revealed by 505–530 band pass filter for Alexa Fluor 488 and by 410–460 band pass filter for DAPI. Images were acquired with the confocal pinhole set to one Airy unit using the same setting (laser power, detector gain, threshold of fluorescence intensity) in all experimental conditions. Three-dimensional reconstructions of Z-slices collected from the top to the bottom of the cells was carried out using Zeiss ZEN Black software as well as quantification analyses. In particular, the mean fluorescence intensities were measured by drawing regions of interest (ROI) around the entire cell as previously described [71] and the values were normalized per cell area. 

### 3.11. Drosophila Stocks 

Flies were reared on standard cornmeal-agar with a 12 h on–off light cycle at 25 °C. Fly stocks used in the current study were obtained from the Bloomington Stock Center (Bloomington, IN, USA): 33,808 w*; P{UAS-HTT.128Q.FL}f27b-8765 w; 438 P{w[+mW.hs]=GawB}elav[C155].

### 3.12. MS3 Treatment and Crosses

During the assays, flies were reared in tubes containing 2 mL of “Assay fly Food” (AF) (2% agar, 10% powdered yeast, 10% sucrose, 0.1% nipagin) or on the same AF supplemented with different MS3 concentrations. Proper volumes of MS3 stock solutions in PBS were added onto the surface of “Assay fly Food” (AF; 2% agar, 10% powdered yeast, 10% sucrose, 0.1% nipagin), and left under gentle agitation for 3 h at r.t. until dryness. PBS was supplemented in equal quantity in all the tested concentrations and in the control food, devoid of aptamer. Expression of polyglutamine-containing hHTT was obtained through the bipartite expression system (UAS)-GAL4 [72], in trans-heterozygous F1 progeny generated by crossing females carrying the pan-neural driver elav-Gal4 to males carrying the UAS HTT128QFL construct at 28 °C. Only F1 adult females elav-GAL4/UAS HTT128QFL were used in this study.

### 3.13. Feeding Assay

To check if the presence of MS3 aptamer in the “Assay fly Food” could affect the feeding of HD and parental flies, the red food dye no. 40 was added to AF medium for each MS3 concentration used [73]. The flies were allowed to feed on the dye-supplemented medium for 1 day, and their abdominal coloring was examined under a stereomicroscope.

### 3.14. Negative Geotaxis Assay and Statistics

Negative geotaxis assay was carried out as previously described [64]. Briefly, a group of 20 sex-matched flies were placed in a graduated empty plastic vial (18 × 2.5 cm) and allowed to recover for 30 min. Negative geotaxis was measured by recording the number of flies that climbed above the 9 cm mark within 20 s after a tap down of the flies to the bottom of the vial. This assay was repeated for the same group twice, allowing for a 1 min rest period between each trial. The number of flies per group that passed the 9 cm mark was recorded as a percentage of total flies. The number of flies per group in each segment was also recorded to calculate the average climbing height. For each condition, 3 groups of 20 flies each were tested in the marked tube in three independent experiments, and the data were expressed as an average of the replicates (*n* = 180).

Statistical analysis was performed using one-way analysis of variance (ANOVA) followed by Dunnett’s multiple comparisons test, in Graph Pad Prism 9.

## 4. Conclusions

Mutation in the structure of huntingtin protein has emerged as the main player in the progression of HD. Therefore, the mutant protein is considered a privileged target of many cutting-edge pharmacological strategies to fight HD, a significant neurodegenerative disease for which, currently, only limited, palliative treatments are available. Among the potential candidate drugs developed to specifically bind the mutant protein, a set of guanine-rich aptamers have recently been evolved and analyzed in a variety of biochemical experiments which demonstrate their capability of binding to a mutant huntingtin protein, with a 78-residue polyglutamine tract expansion, and influence its activity [41].

Aiming at profitably contributing to this research field, we have here provided a detailed biophysical characterization of the best aptamer in the series, named MS3. Our data proved that this aptamer adopts a very stable, parallel G-quadruplex structure, as determined by UV, CD, and DSC measurements, and shows high resistance to nuclease digestion in pseudo-physiological solutions. The in vitro characterization picture of this anti-huntingtin aptamer has been completed by confocal microscope analyses. These experiments proved that fluorescein-labelled MS3 is rapidly internalized in both non-neuronal HeLa and neuronal SH-SY5Y cells in a dose-dependent process and persists in the examined neuronal cells for a long time (up to 72 h), not causing evidence of general cytotoxicity. This result is particularly relevant, demonstrating the feasibility of its in vivo use, even in the absence of transfecting agents. Finally, using a well-established *Drosophila melanogaster* model for Huntington’s disease (Q128HD-FL), which expresses the mutated form of human huntingtin, the neuronal function improved after MS3 treatment, definitively proving the in vivo efficacy of this aptamer.

The intriguing properties shown by the MS3 aptamer open new valuable perspectives in the therapeutic approaches for Huntington’s disease based on specifically targeting mutant huntingtin.

## Figures and Tables

**Table 1:** Thermodynamic parameters of MS3 determined by DSC and CD measurements in the selected Na^+^-containing buffer (10 mM NaH_2_PO_4_/Na_2_HPO_4_, 90 mM NaCl solution, pH 7.0).

	T_m_ (°C)	Δ_exp_H° (kJ mol^−1^)	Δ_vH_H° (kJ mol^−1^)	Δ_exp_S° (kJ K^−1^ mol^−1^)	Δ_exp_G° (kJ mol^−1^) at 310 K
MS3	53	205	175 167 ^1^	0.627	10.6

^1^ From CD measurements. The error on T_m_ determination is ±1.0 °C and on the other thermodynamic parameters is ±10%.

## Supplementary Materials

The following supporting information can be downloaded at: https://www.mdpi.com/article/10.3390/ijms23094804/s1.
