# Supplementary material for: Fighting the Huntington’s Disease with a G-Quadruplex-Forming Aptamer Specifically Binding to Mutant Huntingtin Protein: Biophysical Characterization, In Vitro and In Vivo Studies
Source: Int J Mol Sci. 2022 Apr 27;23(9):4804. doi: 10.3390/ijms23094804 (PMC9101412; doi:10.3390/ijms23094804)
Supplement: Supplementary file 1 [file ijms-23-04804-s001.zip › ijms-1691932-supplementary.pdf]

Supplementary Materials

to the manuscript

Fighting the Huntington's disease with a G-quadruplex-forming aptamer specifically binding to mutant huntingtin protein: biophysical characterization, in vitro and in vivo studies

Claudia Riccardi,[a,ǂ] Federica D'Aria,[b,ǂ] Filomena Anna Digilio,[c] Maria Rosaria Carillo,[d] Jussara Amato,[b] Dominga Fasano,[e,f] Laura De Rosa,[e] Simona Paladino,[e] Mariarosa Anna Beatrice Melone,[f,g] Daniela Montesarchio,[a,*] Concetta Giancola[b,*]

[a]*Department of Chemical Sciences, University of Naples Federico II, 80126 Napoli, Italy*
[b]*Department of Pharmacy, University of Naples Federico II, 80131 Napoli, Italy*
[c]*Research Institute on Terrestrial Ecosystems (IRET), UOS Naples-CNR, 80131 Napoli, Italy*
[d]*Department of Experimental Medicine, University of Campania Luigi Vanvitelli, Naples, Italy*
[e]*Department of Molecular Medicine and Medical Biotechnology, University of Naples Federico II, 80131 Napoli, Italy*
[f]*Department of Advanced Medical and Surgical Sciences, 2nd Division of Neurology, Center for Rare Diseases and InterUniversity Center for Research in Neurosciences, University of Campania Luigi Vanvitelli, 80131 Napoli, Italy*
[g]*Sbarro Institute for Cancer Research and Molecular Medicine, Center for Biotechnology, Temple University, Philadelphia, PA 19122-6078, USA*

[ǂ]These authors equally contributed to this work.
*Authors to whom correspondence should be addressed.

Table of contents

Figure S1. TDS profiles of MS3 **pag. S1**

Table S1. TDS factor analysis **pag. S2**

Figure S2. Prediction of G4 structure topologies adopted by MS3 determined by SVD analysis **pag. S3**

Figure S3. Three-dimensional melting curves for MS3 **pag. S4**

Table S2. Singular Value Decomposition of 3D Melting Data **pag. S5**

Figure S4. Morphological analysis of untreated and treated cells **pag. S6**

References **pag. S7**

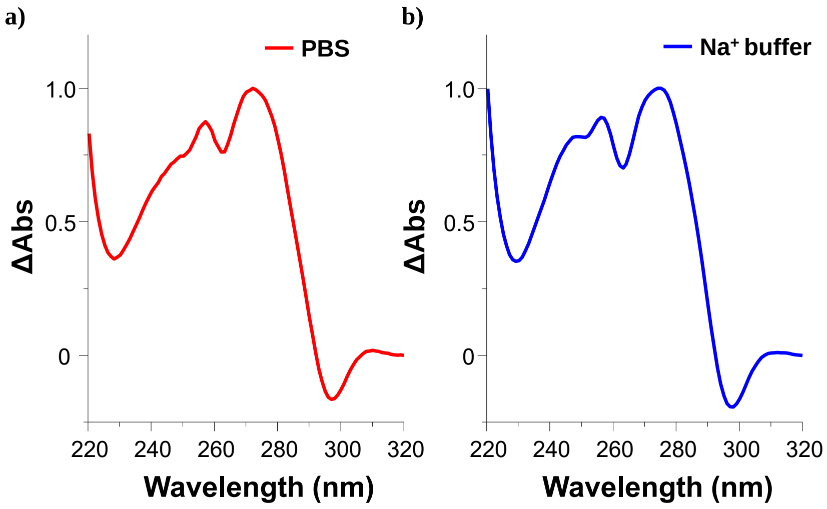

Figure S1. Normalized TDS profiles of MS3 at 2 μM concentration in the selected PBS (**a**) and Na^+ (**b**) buffer solutions. TDS profiles were obtained by subtraction of each UV spectrum recorded at 5 °C from the corresponding one registered at 100 °C.

Table S1. TDS factors, *i.e.* ratios between absorbance values at different wavelengths as calculated from normalized TDS spectra for MS3 in both the selected phosphate buffer solutions, according to literature protocols.(1)

	TDS factors		
	$\Delta A_{240}/\Delta A_{295}$	$\Delta A_{255}/\Delta A_{295}$	$\Delta A_{275}/\Delta A_{295}$
**PBS**	**4.5**	**6.3**	**7.2**
**Na^+ buffer**	**4.3**	**5.8**	**6.6**

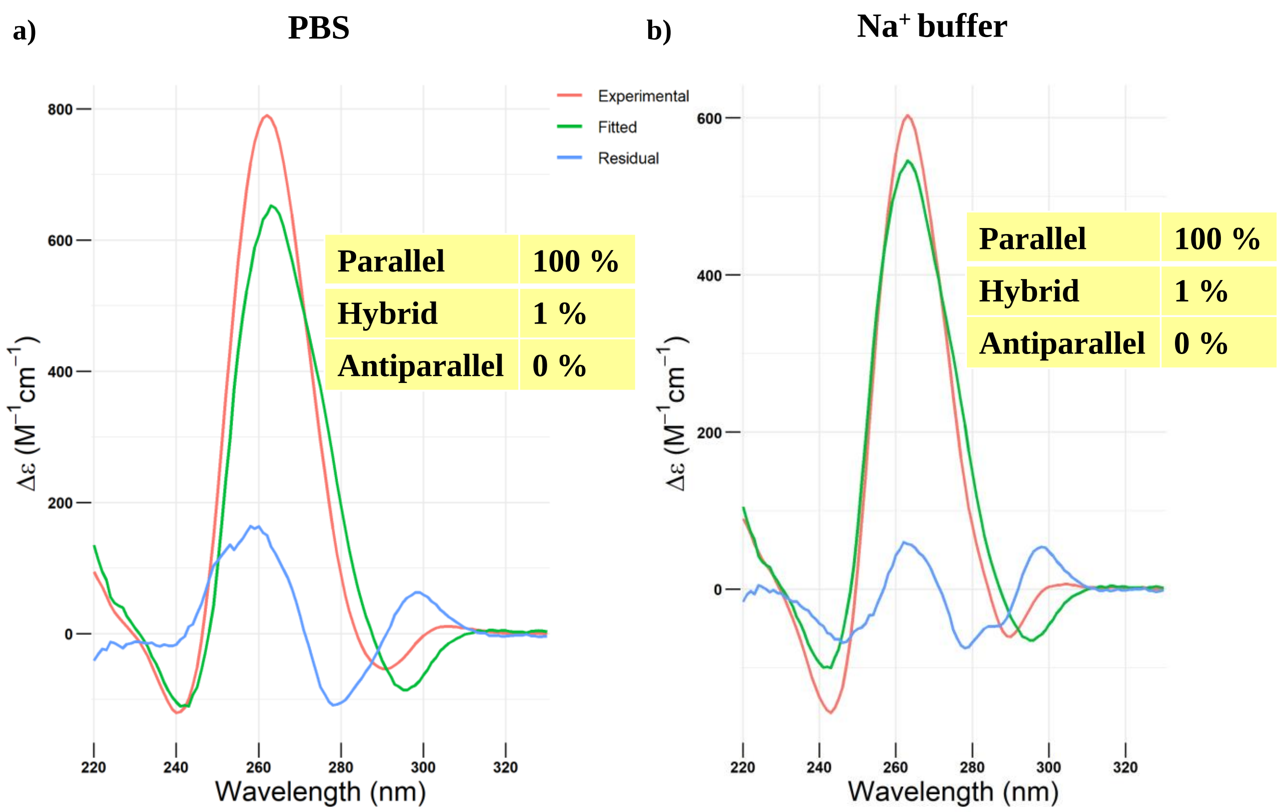

Figure S2. Prediction of the relative abundance of the different G4 topologies adopted by MS3 as obtained by singular value decomposition (SVD) analysis of the CD spectra recorded in both the selected buffer solutions, performed by exploiting the software developed by del Villar-Guerra *et al.*(2) Deviations from 100% (± 1%) are due to significant digits approximation of the values originally obtained by the simulations.

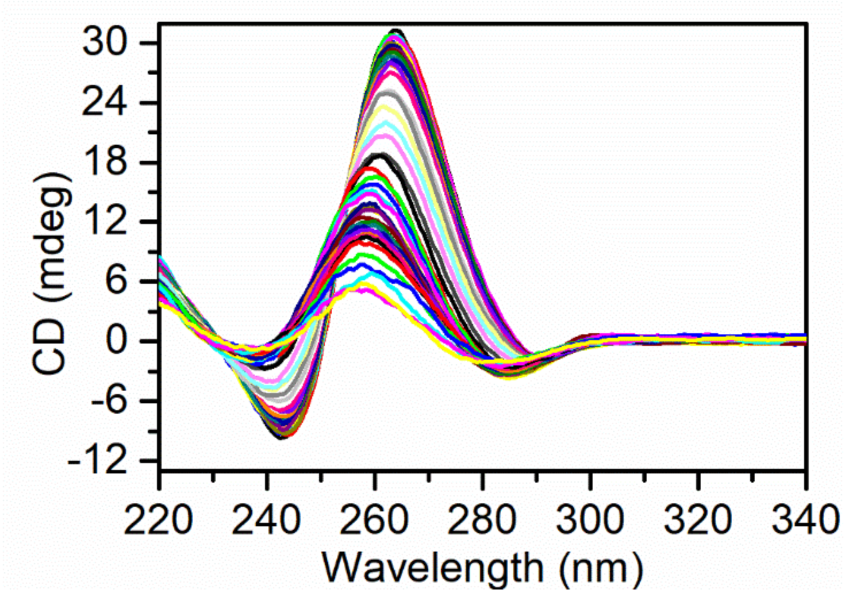

Figure S3. Three-dimensional melting curves obtained for MS3 in the Na^{+}-containing buffer.

Table S2. Results from Singular Value Decomposition of 3D CD-melting data.

	Autocorrelation	
Singular Values	U matrix	V matrix
806.097	0.9973	0.9764
182.412	0.9966	0.9780
20.517	0.9512	0.7458
15.135	0.9911	0.0954
7.529	0.9847	0.2193
7.022	0.9724	-0.0021
6.377	0.9742	0.0387
5.685	0.9743	-0.1931
4.868	0.9244	0.1343
4.411	0.9760	0.2570

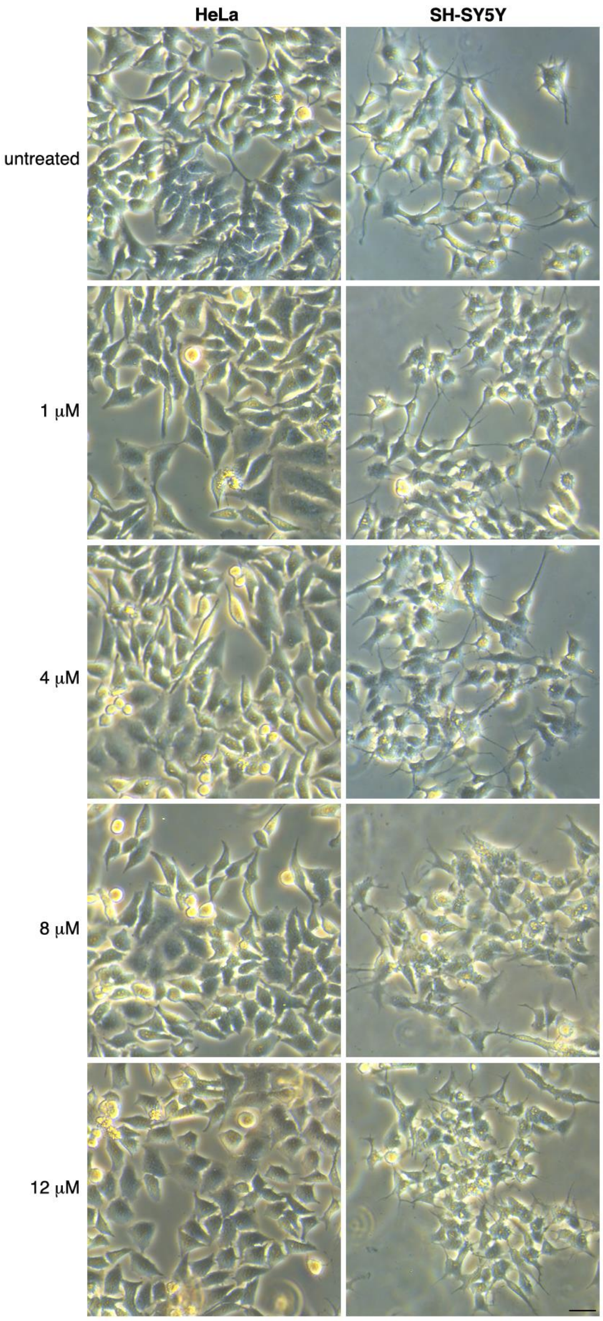

Figure S4. Morphological analysis of untreated and treated HeLa and SH-SY5Y cells. Cells were incubated with FITC-MS3 for 24 h at the indicated concentrations; representative phase-contrast images are shown. Scale bars, 12 μm.

References

1. Karsisiotis,A.I., Hessari,N.M.A., Novellino,E., Spada,G.P., Randazzo,A. and Webba da Silva,M. (2011) Topological characterization of nucleic acid G-quadruplexes by UV absorption and circular dichroism. *Angew. Chem. - Int. Ed. Eng.*, **50**, 10645–10648.
2. Del Villar-Guerra,R., Trent,J.O. and Chaires,J.B. (2018) G-quadruplex secondary structure obtained from circular dichroism spectroscopy. *Angew. Chem. - Int. Ed. Eng.*, **57**, 7171–7175.
